# Identification of a Shared Microbiomic and Metabolomic Profile in Systemic Autoimmune Diseases

**DOI:** 10.3390/jcm8091291

**Published:** 2019-08-23

**Authors:** Chiara Bellocchi, Álvaro Fernández-Ochoa, Gaia Montanelli, Barbara Vigone, Alessandro Santaniello, Rosa Quirantes-Piné, Isabel Borrás-Linares, Maria Gerosa, Carolina Artusi, Roberta Gualtierotti, Antonio Segura-Carrettero, Marta E. Alarcón-Riquelme, Lorenzo Beretta

**Affiliations:** 1 Referral Center for Systemic Autoimmune Diseases, Fondazione IRCCS Ca’ Granda Ospedale Maggiore Policlinico di Milano, 20122 Milan, Italy; 2 Department of Clinical Sciences and Community Health, University of Milan, 20122 Milan, Italy; 3 Department of Analytical Chemistry, University of Granada, 18016 Granada, Spain; 4 Research and Development of Functional Food Centre (CIDAF), Health Science Technological Park, 18016 Granada, Spain; 5 Centre for Genomics and Oncological Research (GENYO), Pfizer-University of Granada-Andalusian Regional Government, 18016 Granada, Spain; 6 Institute for Environmental Medicine, Karolinska Institutet, 40225 Stockholm, Sweden

**Keywords:** microbiomic, metabolomics, systemic autoimmune diseases, systemic lupus erythematosus, Sjögren’s syndrome, primary anti-phosholipid syndrome, undifferentiated connective tissue diseases

## Abstract

Dysbiosis has been described in systemic autoimmune diseases (SADs), including systemic lupus erythematosus (SLE), Sjögren’s syndrome (SjS), and primary anti-phosholipid syndrome (PAPS), however the biological implications of these associations are often elusive. Stool and plasma samples from 114 subjects, including in SLE (*n* = 27), SjS (*n* = 23), PAPs (*n* = 11) and undifferentiated connective tissue (UCTD, *n* = 26) patients, and geographically-matched healthy controls (HCs, *n* = 27), were collected for microbiome (16s rRNA gene sequencing) and metabolome (high-performance liquid chromatography coupled to mass spectrometry) analysis to identify shared characteristics across diseases. Out of 130 identified microbial genera, a subset of 29 bacteria was able to differentiate study groups (area under receiver operating characteristics (AUROC) = 0.730 ± 0.025). A fair classification was obtained with a subset of 41 metabolic peaks out of 254 (AUROC = 0.748 ± 0.021). In both models, HCs were well separated from SADs, while UCTD largely overlapped with the other diseases. In all of the SADs pro-tolerogenic bacteria were reduced, while pathobiont genera were increased. Metabolic alterations included two clusters comprised of: (a) members of the acylcarnitine family, positively correlating with a Prevotella-enriched cluster and negatively correlating with a butyrate-producing bacteria-enriched cluster; and (b) phospholipids, negatively correlating with butyrate-producing bacteria. These findings demonstrate a strong interaction between intestinal microbiota and metabolic function in patients with SADs.

## 1. Introduction

Gut microbiota contributes to immune system development and to immune defense processes involving both innate and adaptive immunity [1,2,3,4]. Specific gut bacterial phylotypes are involved in immune tolerance processes, including the activation of T regulatory cells through the production of short chain fatty acid (SCFA) [5,6,7].

Investigations of the intestinal microbiome in patients with systemic autoimmune diseases (SADs) have demonstrated a reduction of pro-tolerogenic bacterial species [8,9,10,11]. However, the functional consequences of dysbiosis in SADs remain elusive. Few studies have investigated the intestinal microbial profile in SADs from a multi-omic perspective by integrating microbiome and metabolome data to provide insight into the functional characteristics of microbiota in these disease states [11,12,13].

A microbiome analysis in patients with systemic lupus erythematosus (SLE) showed a reduction of intestinal microbial biodiversity and an altered SCFA production [9]. In mouse models of SLE, the administration of antibiotics to counteract dysbiosis ameliorated SLE-related symptoms [14]. Recently, *Enterococcus gallinarum* was increased in SLE mice and found to be associated with an increased gut epithelium permeability [15]. Metabolomic signatures have been investigated in a limited number of SLE studies. The most consistent alterations were found in unsaturated fatty acids and acyl-carnitines, as well as in phospholipid metabolites [16,17].

Very few studies have explored gut microbiota or serum metabolites in Sjögren’s syndrome (SjS) [18,19]. In a study conducted on 42 SjS and 35 healthy controls, patients with SjS had an increased prevalence of gut dysbiosis compared with controls and the extent of dysbiosis was correlated with disease severity [20]. Among the metabolomic studies in SjS performed in comparison with SLE or RA subjects, no definite conclusion on SjS metabolic fingerprints could be drawn from these works [17,21].

Various hypotheses on the pathogenesis of primary antiphospholipid syndromes (PAPs) have been formulated. The potential molecular cross-reactivity between autoantigens and repeated sequences of bacterial peptides has been hypothesized to stimulate T-cell mediated responses and B cell production of specific PAPs auto-antibodies. Intestinal microbiota could potentially serve as the source of bacteria stimulating a chronic systemic inflammatory response [22]. To our knowledge, only one study has been performed investigating the intestinal microbiota in PAPS patients, and this study found a reduction of Slackia strains (which are able to produce phospholipids including cardiolipin) and of Butyricides (pro-tolerogenic bacteria) [23]. The only metabolomic analysis conducted so far identified abnormalities associated with the metabolism of methyl group donors, ketone bodies, amino acids, and PAPS [24].

To date, there are still no clear guidelines and no recent studies on undifferentiated connective tissue disease (UCTD) [25] that examine the pathogenic aspects of this condition. Consequently, neither intestinal microbiota nor plasma metabolome analysis have been performed in this group of patients.

The present study investigated the intestinal microbiome and plasma metabolome in patients with distinct SADs, including SLE, SjS, PAPS, and UCTD. We hypothesized that SADs may share common microbiota features across disease states that contrast with microbiota features of healthy controls (HCs).

## 2. Methods

### 2.1. Patients and Controls

A total of 114 subjects were enrolled in the study, including 27 SLE, 23 SjS, 11 PAPs, and 26 UCTD patients, plus 27 ethnically-, age-, and sex-matched HCs. All of the patients were recruited at the Referral Center for Systemic Autoimmune Diseases, Fondazione Istituto Ricerca a Carattere Scientifico (IRCCS) Ca’ Granda Ospedale Maggiore Policlinico of Milan, and at the Department of Rheumatology, Azienda Scocio Sanitaria Territoriale (ASST) Istituto Gaetano Pini and Centro Traumatologico Ortopedico (CTO) of Milan. SLE patients fulfilled the 1997 update of 1982 American College of Rheumatology criteria [26]. SjS patients fulfilled the American European Consensus Group (AECG) SjS classification criteria [27]. PAPs patients fulfilled the international consensus statement on an update of the classification criteria for definite PAPs [28] and UCTD were defined as those subjects with clinical features of SADs fulfilling none of the above criteria nor any other SADs criteria for at least 2 years, plus the presence of antinuclear antibodies (ANA) ≥ 1:160 with or without SAD-specific autoantibodies.

Clinical and laboratory characteristics of patients were recorded in the PRECISESADS case report form and later extracted for the present study. Autoantibodies were centrally determined at the Laboratory of Immunology and Immunotherapy of the Université Bretagne Occidentale (UBO) via a chemiluminescence autoantibody screening strategy using the IDS-iSYS immunoanalyzer (Boldon, UK) and coated magnetic particles (solid phase) coupled with autoantigens (Technogenetics, Milan, Italy). Polyclonal human rheumatoid factors (RF), complement C3c, C4, were measured using a turbidimetric immunoassay (SPAPLUS^®^ analyzer, The Binding Site, Grassobbio, Italy).

Our research is ancillary to the PRECISESADS project (https://clinicaltrials.gov/ct2/show/NCT02890121) that was approved by the local ethics committee (Comitato Etico Area 2, Milano; approval no. 425bis dated Nov 19, 2014, and no. 671_2018 dated Sep 19, 2018).

### 2.2. Collection and Storage of Samples

Patients donated stool samples and within 24 hours of the stool collection the plasma samples were obtained for metabolomic analysis and for autoantibody determination (see above). None of the patients were treated with antibiotics or probiotics within 4 weeks of the collection of fecal samples, which were frozen at −20 °C until delivery to the laboratory of probiogenomics of Parma University for processing and analysis. Plasma samples were frozen at −80 °C until delivery to the University of Granada for metabolomic analysis.

### 2.3. 16S rRNA Gene-Sequencing

DNA was extracted from each stool sample using DNA extraction using the QIAamp DNA Stool Mini kit following the manufacturer’s instructions (Qiagen).

Partial 16S rRNA bacterial gene sequences were amplified from extracted DNA using primer pair Probio_Uni/Probio_Rev, which target the V3 region of the 16S rRNA gene sequence 16S rRNA. Gene amplification and amplicon checks were carried out as previously described [29]. Notably, the primer pair Probio_Uni/Probio_Rev was specifically developed to maximize coverage and amplification performance of gut bacterial populations. The 16S rRNA gene sequencing was performed using a MiSeq Illumina at the DNA sequencing facility of GenProbio srl [30] according to the protocol previously reported. Following sequencing and demultiplexing, the reads of each sample were filtered to remove low quality and polyclonal sequences and data were exported as fastq files. The fastq files were processed using a custom script based on the QIIME software suite [31]. Paired-end reads pairs were assembled to reconstruct the complete Probio_Uni/Probio_Rev amplicons. Quality control retained those sequences with a 140–400 bp length and mean sequence quality score > 20. Sequences with homopolymers > 7 bp and mismatched primers were omitted. Chimeric sequences were removed with ChimeraSlayer included in the quantitative insights into microbial ecology (QIIME) 2 software suite (http://qiime.org/). To calculate downstream diversity measures, 16S rRNA operational taxonomic units (OTUs) were defined at ≥ 97% sequence homology using uclust and OTUs with < 10 sequences were filtered [32]. All reads were classified to the lowest possible taxonomic rank using QIIME and the SILVA database v. 119 clustered at 97% identity as the reference dataset [33].

### 2.4. Metabolomic Analysis in Plasma

Plasma samples were thawed on ice, then an aliquot of 100 μL of each sample was mixed with 200 μL of methanol:ethanol (50:50, v/v) to remove proteins [34]. The solutions were vortex-mixed and kept at −20 °C for 30 min to achieve an efficient protein precipitation and to avoid possible sample degradation. Subsequently, the samples were centrifuged (4 °C, 10 min, 18,400 × *g*), and the supernatants were evaporated in a vacuum concentrator for 105 min. The dry residue was reconstituted in 100 μL of 0.1% aqueous formic acid:methanol (95:5, v/v), and centrifuged in the same conditions mentioned previously to remove the solid particles. Finally, a 40 μL aliquot was transferred to high performance liquid chromatography (HPLC) vials and stored at −80 °C before their analysis. A quality control (QC) sample was created by mixing equal volumes of each sample (20 μL) and treated as described above.

### 2.5. HPLC-ESI-QTOF-MS Analysis

For the detection of metabolic peaks, a quadrupole-time of flight mass spectrometry analyzer (QTOF-MS) was used coupled to high performance liquid chromatography (HPLC). Specifically, an Agilent 1260 HPLC system (Agilent Technologies, Palo Alto, CA, USA) was used together with an Agilent 6540 Ultra High Definition (UHD) Accurate Mass Q-TOF system (Agilent Technologies, Palo Alto, CA, USA) equipped with a dual-stream Electrospray Ionization (ESI) interface.

The compounds were separated in a C18 reverse phase analytical column (Agilent Zorbax Eclipse Plus, 3.5 μm, 2.1 × 150 mm). The mobile phases were made up of water plus 0.1% formic acid and methanol as solvents A and B, respectively. To obtain an efficient separation of the metabolites, the following gradient of mobile phases was applied: 0 min (A:B, 95/5), 5 min (A:B, 90/10), 15 min (A:B, 15/85), 32–40 min (A:B, 0/100), and 45 min (A:B, 95/5).

A QC sample was analyzed every 5 real samples in order to check the analytical reproducibility and correct possible analytical drifts during the sequence. In addition, a tandem mass spectrometry analysis (MS/MS) of the QC sample was performed to obtain characteristic fragments of molecular species that help in the identification process of candidate metabolites. This experiment was conducted using nitrogen as collision gas with different energy values (10, 20, and 40 eV).

A recursive feature extraction for small molecules was performed on the analyzed samples using the MassHunter Profinder software (B.06.00, Agilent). Peaks were filtered with an intensity threshold of 1000 counts; [M + H]^+^, [M + Na]^+^ and [M + H − H_2_O]^+^ were the considered adducts. The retention times and masses between the different samples were aligned with thresholds of ± 0.25 min and 40 ppm ± 4 mDa, respectively. The exported areas were normalized by the MS total useful signal (MSTUS) [35].

### 2.6. Statistical Analysis

#### 2.6.1. Canonical Analysis of Microbiota

The biodiversity of samples (alpha diversity) was calculated with the Chao1 and Shannon indices using 10 subsampling points for a maximum of 54,890 sequences in order to generate rarefaction curves. Similarities between samples (beta diversity) were calculated by unweighted uniFrac. [36]. Principal coordinate analysis (PCoA) representations of beta diversity were performed using QIIME [31].

For the other analyses, bacteria at the genus taxonomic rank, with an overall abundance >1%, were considered.

#### 2.6.2. Data Mining Classification and Non-Linear Interactions Analysis

To model nonlinear multivariate interactions among variables, several inductive data mining algorithms were used. The overall procedure is described in detail in [11]. Briefly, the study pipeline included a 5-fold nested cross-validation phase (to perform model selection and data filtering) in the context of a repeated (10x) 5-fold cross-validation phase (to assess the robustness and capability of generalization of the selected model). In the model-selection phase, 5 different inductive algorithms were tested: naive Bayes, ada boost (500 runs), random forest (500 runs), extra trees classifiers (500 runs), or voting classifier (soft voting of the 4 previous algorithms). A stochastic hill climbing procedure was used to select the subset of attributes that in the nested cross-validation phase globally optimized one-vs.-one comparisons. The balanced accuracy (BA), which is the average of sensitivity and specificity, was used as the fitness function for modelling and feature selection. Results in the validation phase are expressed as BA, F1 measure (harmonic mean between sensitivity and precision/positive predictive value), and area under receiver operating characteristics (AUROC). BA, F1, and AUROC are presented as the weighted mean of all the possible pairwise comparisons.

In each training fold data were preprocessed, standardizing data values and removing 5% of the outliers via the isolation forest method (500 trees). For the microbiome dataset, genera with values equal to zero in > 95% of the cases were removed.

To graphically represent multidimensional results we used the FreeViz method [37]. This method selects, via a gradient descent algorithm, the representation that optimizes the compactness and separation of instances of the same class, as assessed by average silhouette scores [38].

A subanalysis comparing the overall anti Ro60/SSA positive versus anti Ro60/SSA negative patients was conducted with the same procedures described above.

#### 2.6.3. Post-Hoc Statistical Analysis on Classification Data

Selected bacterial genera and metabolomic peaks were compared between study groups by Kruskal–Wallis tests and if significant after false discovery rate (FDR) correction at the nominal 0.05 alpha level, the Dunn test was performed for post-hoc analysis.

#### 2.6.4. Cross-Correlation Analysis

In patients with SADs, microbial genera (G) were correlated with metabolites (M) correcting for potential confounding variables at the time of sampling: age, the use of hydroxycloroquine (yes/no), the use of prednisone > 5 mg/day (yes/no), and the use of immunosuppressants (any type, yes/no). To this end, a partial correlation analysis was performed considering plasma metabolites as dependent (Y) variable, microbial genera as independent variable (Z), and the confounding variables as covariates (X). To test the null hypothesis of no relationship between Y and Z over and above any relationship of Y with X, we calculated the Pearson’s r coefficient using the residuals RY|X (residuals of regression equation of Y on X alone) and RZ|X (residuals of regression equation of Z on X alone). The procedure was iteratively repeated to produce a G x M cross-correlation matrix. The significance of matrix entries were calculated and corrected for multiple testing using a stepwise pMin procedure randomly permuting raw data (Y variable); 10,000 random runs were used to this end [39,40].

Partial r values in the G x M cross-correlation matrix were used to cluster genera and metabolites by means of the hierarchical clustering and Wards linkage methods and manually inspecting the cluster tree. The average (centroid) relative abundances and peak areas from clustered genera and metabolites were calculated for each individual; centroids were then used to build a simplified cross-correlation matrix. Correlation data were corrected for confounding variables taking into account multiple testing as described above.

For all of the analyses, custom codes written in python by L. B. built on top of the scikit-learn modules [41] were used; codes are available upon request. Clusters were built with the Orange data mining suite (https://orange.biolab.si/).

## 3. Results

Clinical and demographic characteristics of the study participants are reported in Table 1. SADs patients were mostly female, whereas a slightly higher proportion of males was observed in HCs. Physician global assessment scale was similar in the 4 groups of SADs, with a slight increase in PAPs patients. All four disease groups had an average prednisone intake of less than 10 mg per day. Prevalent clinical features and autoantibody specificities were consistent with the baseline diagnosis.

### 3.1. Microbiomic Analysis

**Figure 1:**
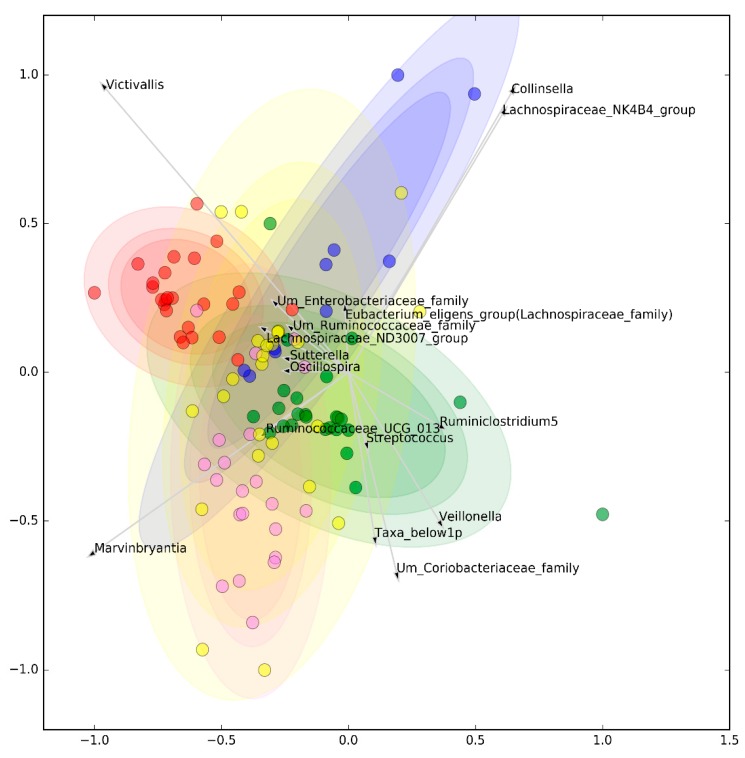
Clustering of selected microbiome genera. Visual representation by FreeViz projections of selected microbiome genera that best maximize the global pairwise goodness of fit (mean of all the possible one-vs.-one comparisons). The graphical representation is optimized to maximize the compactness and separation of clusters as measured by silhouette scores. Note: Red = healthy controls; purple = systemic lupus erythematosus; green = Sjogren’s syndrome; blue = primary antiphospholipid syndrome; yellow = undifferentiated connective tissue disease. Shaded areas indicate 99%, 95%, and 90% confidence ellipse intervals (from darkest to lightest).

After performing microbiome quality filtering (Supplementary File 1), our alpha diversity analysis found no differences either in richness or in evenness of taxa among the study groups (Supplementary Figure S1). Similarly, principal coordinate analysis (PCoA) analysis of beta diversity could not separate any disease from the others, albeit HCs tended to cluster apart from SADs (Supplementary Figure S2). On the contrary, classification algorithms discriminated the different diseases with AUROC = 0.727 ± 0.034, F1 = 0.704 ± 0.024, and BA = 0.654 ± 0.018 when all of the genera were considered. A similar discrimination could be obtained after feature selection, highlighting the importance of selecting a subset of bacteria in differentiating the different classes: AUROC = 0.730 ± 0.025, F1 = 0.717 ± 0.034, and BA = 0.663 ± 0.031. The final model included 29 genera (Supplementary Table S1) and almost completely separated HCs from SADs, as shown in Figure 1; the capability of this subset of bacteria to differentiate the SADs and HCs (pairwise comparisons) according to the different classification metrics is represented in Supplementary Figure S3. According to FreeViz representation, HCs emerged as an isolated cluster, while UCTD patients had the highest degree of overlap with the other autoimmune diseases.

Univariate analysis showed that the abundance of some genera was statistically different in SADs compared to HCs. Namely, the abundance of *Bifidobacterium, Ruminiclostridium, Streptococcus,* U. m. of *Coriobacteriaceae* family, U. m. of *Enterobacteriaceae* family, and *Collinsella* was significantly lower in the HCs group compared to the other groups (Supplementary Figure S4), while the abundance of *Lachnoclostridium, Lachnospira*, and *Sutterella* was significantly increased in HCs compared to SLE, SjS, PAPs, and UCTD (Supplementary Figure S5).

The subanalysis of microbiome comparing anti Ro60/SSA positive versus anti Ro60/SSA negative patients did not show significant results (BA and AUROC < 0.6).

### 3.2. Metabolomic Analysis

**Figure 2:**
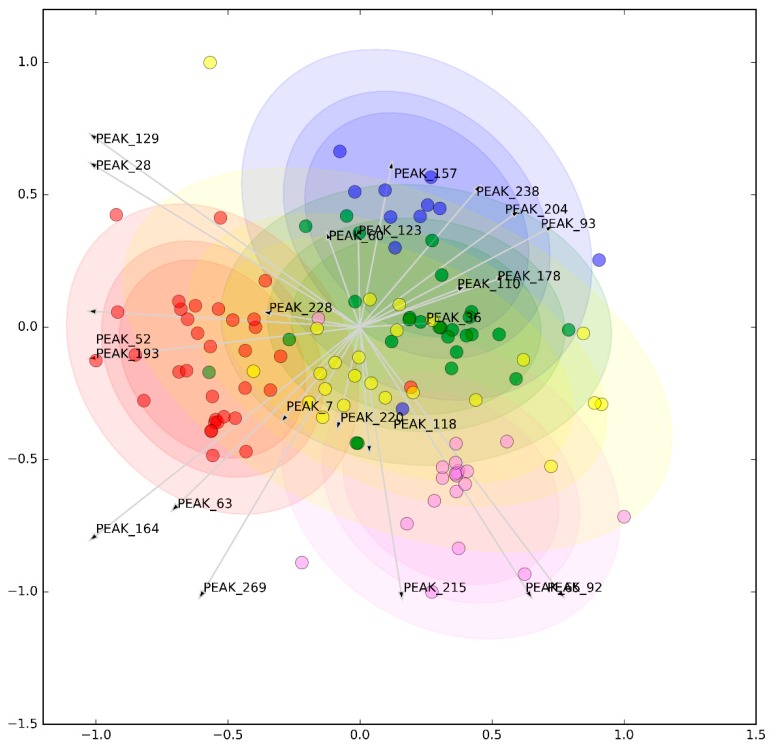
Clustering of selected metabolites. Freeviz representations of selected metabolites (see legend to Figure 1 for details).

Feature selection in our metabolomic analysis slightly reduced the original fit of the complete model. A reduced model comprised of 41 metabolites was capable of performing a fair joint pairwise classification of diseases with an AUROC = 0.748 ± 0.021 (vs. 0.766 ± 0.015 of the full model), F1 = 0.684 ± 0.015 (vs. 0.702 ± 0.023), and BA = 0.671 ± 0.013 (vs. 0.674 ± 0.02). The selected significant peaks are detailed in Supplementary Table S2, while their multidimensional representation is illustrated in Figure 2. The metabolites were identified by the comparison of the MS results (mass exact, isotopic distribution, and MS/MS fragments) and the information from the metabolomic databases. However, despite the efforts in the identification, a number of peaks remained unknown because this stage is currently the bottleneck in the field of metabolomics [42]. Pairwise comparisons are illustrated in Supplementary Figure S6, in which select metabolites could discriminate HCs from SADs, while their performance in SAD-to-SAD comparisons was less remarkable.

A number of peaks were differentially distributed in the analyzed groups according to univariate statistical analysis, as highlighted in Supplementary Table S2. Not surprisingly, most of the differences were due to reduced or increased normalized peak area values in HCs compared to one or more disease groups.

The analysis in anti-Ro60/SSA positive versus anti-Ro60/SSA negative patients showed that the two groups could weakly, albeit non-substantially be distinguished from the metabolic point of view (average AUROC = 0.689; BA = 0.569).

### 3.3. Cross-Correlation Analysis

**Figure 3:**
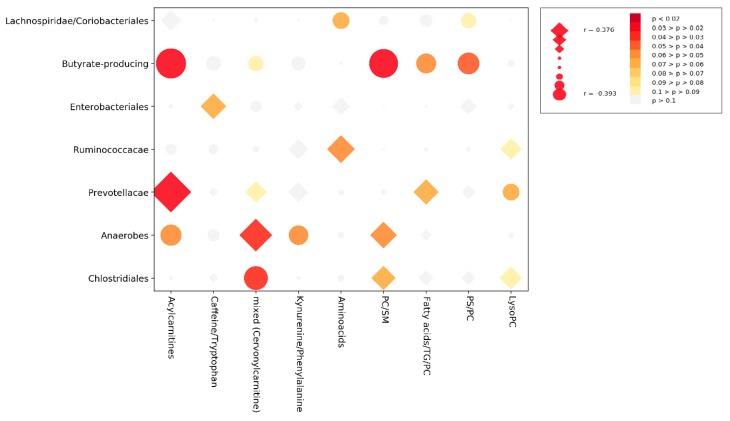
Simplified cross-correlation. Partial correlation analysis of aggregated microbiome and metabolome data after correction for confounding variables (age, hydroxycloroquine, steroid and immunosuppressant use). Aggregated clusters are calculated from the full correlation matrix shown in Supplementary Figure S7, as described in the methods and manually annotated. Note: Diamonds, positive partial correlations; circles, negative partial correlations; *p* values assessed after permutation testing.

Supplementary Figure S7 represents the cross-correlation matrix corrected for age and concurrent therapies among microbial genera and metabolites; correlations significant at the 0.05 threshold after permutation test and with an absolute Pearson’s r > 0.3 are highlighted. According to partial correlation coefficients, we could distinguish 7 bacterial and 9 metabolite clusters. Data aggregation of individual data within these clusters yielded the simplified cross-correlation matrix illustrated in Figure 3. Attempts were made to identify metabolites within the clusters yielding the most significant partial correlation values. We could observe an enrichment of specific metabolites within each cluster: Cluster 1: Acylcarnitines; Cluster 2: Caffeine/Tryptophan; Cluster 3: mixed; Cluster 4: Kynurenine/Phenylalanine; Cluster 5: Aminoacids; Cluster 6: phosphatidylcholine (PC) and sphingomyelin (SM); Cluster 7: Fatty acids and tryglicerides (TG) and PC; Cluster 8: phosphatidylserine (PS) and PC; Cluster 9: LysoPC.

## 4. Discussion

To our knowledge, this is the first study to investigate and compare the gut microbiota and plasma metabolome profiles of 4 distinct SADs (SLE, SjS, UCTD and PAPS). Instead of focusing on specific alterations that may explain putative differences between HCs and single diseases, we tried to discover a common set of alterations jointly capable of discriminating SADs from HCs. Albeit with individual differences, our findings indicate that SADs share similar etiologic factors and pathophysiological mechanisms similar to the common genetic background or similar epigenetic processes that have been described in these disease states [43]. Among the analyzed SADs, the microbiota and metabolome profiles of patients with UCTD overlapped with other disease groups, likely due to the fact patients with this phenotype have nonspecific clinical features of the other SADs under study [44].

In terms of specific microbial findings, we observed an increased relative abundance of *Bifidobacterium, Ruminiclostridium, Streptococcus,* U. m. of *Coriobacteriaceae* family, and U. m. of *Enterobacteriaceae* family in all of the SADs compared to HCs. *Bifidobacterium* is known to be increased in active phase of inflammatory bowel disease [45]; similarly, *Streptococcus* has been associated with inflammatory intestinal conditions [46]. Taxa belonging to the order of *Clostridiales* (i.e., *Ruminiclostridium*) have been found in lower abundance in Crohn’s disease. In murine models of RA, the expansion of *Clostridiales* was correlated with transcription of pro-inflammatory cytokines (Th1/Th17) [47]. Similarly, a high relative abundance of *Streptococcus* has been associated with relapsing-remitting multiple sclerosis and increased Th17 cell frequency [48]. Interestingly, in our SLE and PAPS subjects, we noticed an increased relative abundance of *Collinsella*, another taxon, which was increased in RA patients and correlated with IL-17a [49].

Conversely, we found a reduction of *Lachnoclostridium, Lachnospira*, and *Sutterella* in all SADs groups compared to HCs. *Lachnoclostridium* and *Lachnospira* are part of *Lachnospiraceae*, a family of bacteria capable of producing SCFA (such as butyric acid) with a highly pro-regulatory, tolerogenic role on immune functions [50]. Our results are, thus, aligned with several previous observations of a depletion in tolerogenic bacteria in patients affected by SADs [9,10,47].

Consistent with our microbiomic findings, the metabolomic analysis revealed that SADs had unique metabolomic features distinguishing them from HCs. A relatively small subset of peaks (*n* = 41) was able to maximize the discrimination among groups. Despite the observation that some metabolites were differentially represented in selected SADs compared to HCs, a unifying discriminative pattern could not be identified. These findings highlight the complex interaction among metabolites in SADs and underline that several metabolic pathways may be non-linearly involved in their phenotypic expression. Our results are in accordance with Bengtsson et al., where very few metabolites withstand statistical correction, yet provide a good separation between SjS and HCs or SLE, and to a lesser extent between SLE and HCs when jointly considered [17]. Of interest, among the annotated peaks in our reduced classification model we could identify several members of the acylcarnitine family, suggesting a deregulation of β-oxidation processes in SADs, as already suggested in RA, SLE, or SjS [17,51,52,53,54].

As a confirmation of the concordant behavior of the microbiomic and the metabolomic profile observed by our data mining analysis, we observed a moderate-to-strong correlation among selected individual metabolic peaks and bacterial genera. Although a causal relationship cannot be established by this kind of analysis, we could extract some metabolite and bacterial clusters that correlated with each other. In particular, a cluster enriched by acylcarnitine metabolites was significantly directly correlated with a group mostly represented by *Prevotella* genera. In support of this correlation, a dietary intake of carnitine has been implicated in trimethylamine N-oxide (TMAO) production, an amine oxide obtained from carnitine, betaine, and choline through gut microbiota metabolism. Bacterial families implicated in TMAO production include *Enterobacteriaceae* and *Prevotellaceae* [55]. Koeth et al. found a significant increase in plasma TMAO related to an enterotype rich in *Prevotella* and peculiar of omnivore subjects [56].

On the contrary, an inverse correlation between the acylcarnitine family cluster and a cluster of bacteria mainly composed of butyrate-producing strains was found. This cluster includes *Anaerostipes, Subdoligranulum, Intestinimonas* and *Flavonifractor* [57], *Eisenbergiella* [58], *Megamonas* [59], and *Cloacibacillusg* [60]. Butyrate-producing bacteria are immunologically pro-tolerogenic strains that metabolize SCFA mostly obtained from fruits and vegetables, while on the contrary carnitine intake from diet derives mostly from red meat. Additionally, we also found an inverse correlation between a PC-enriched cluster and the butyrate-producing, bacteria-enriched cluster. In complex experiments and putative explanatory models, butyrate was shown to upregulate cytosolic phospholipase A2 (PLA2) activity in macrophages and secretory PLA2 activity in adipocytes and macrophages to ultimately suppress lipolysis [61]. As lipolysis participates in inflammatory signaling processes, we may hypothesize that in SADs the (loss) of butyrate-producing bacteria may influence inflammation, disrupting PC and lysoPC homeostasis [62,63].

While a number of results from our study are intriguing, we shall acknowledge some drawbacks of our work. Mechanistic studies on animal models would be a fundamental integration of the observed data to better define the functional role of identified bacteria. Moreover, even if the overall number of enrolled patients is satisfactory and unprecedented in SADs studies, there is a lack of homogeneity because PAPs are underrepresented compared to the other. Geographical implications on microbiome and metabolome results should also be taken into account and we cannot guarantee that our findings are generalizable to genetically diverse populations with different lifestyles and dietary habits. Lastly, a thorough analysis comparing patents with different autoantibody subsets could have provided additional microbiomic and metabolomic information. It is known that the presence of specific autoantibodies may be more informative than the clinical phenotypic stratification [64,65] and may have omic implications as well [66,67]. Unfortunately, despite our attempts, the subanalysis based on Ro60/SSA specificity yielded inconclusive results, most likely due to the inadequate number of patients and to the unbalanced nature of data. As such, more focused and powerful studies are required to solve this issue.

## 5. Conclusions

Our findings indicate that SLE, UCTD, SjS, and PAPS patients share common intestinal microbiome and metabolomic profiles. Although specific causative explanations cannot be made, a correlation between microbial genera and metabolites affirm the hypothesis that an interaction between intestinal microbiota and metabolic function exists. These data indirectly support the notion that modulation of microbiota, either by diet or by other means, may be a potential strategy to tackle the systemic consequences of dysbiosis, including inflammation and autoimmunity [64,65]. The optimal strategy to modulate dysbiosis in SADs remains elusive, yet the study of the metabolome appears to be a valuable option to monitor the effect of such an intervention and warrants further research.

## Figures and Tables

**Table 1:** Demographic and clinical characteristics of study participants.

	SLE (*n* = 27)	SjS (*n* = 23)	PAPS (*n* = 11)	UCTD (*n* = 26)	HC (*n* = 27)
Age, mean (SD)	47.70 (16.55)	65.91 (12.72)	40.36 (6.17)	52.23 (12.01)	52.47 (9.96)
Females, *n* (%)	24 (88.9)	22 (95.65)	8 (72.72)	23 (88.46)	20 (74.07)
Disease duration years, mean (SD)	15.16 (10.92)	9.95 (9.38)	11.17 (5.92)	9.43 (5.09)	-
AutoAb profile, *n* (%)					
ANA	27 (100)	20 (86.97)	2 (16.67)	26 (100)	
Anti-Ro 60/SSA	4 (14.81)	15 (65.22)	0 (0)	3 (11.53)	
Anti-La/SSB	1 (3.70)	10 (43.48)	0	0	
Anti dsDNA	13 (48.14)	0 (0)	2 (18.18)	5 (19.23)	
Anti Sm	2 (7.4)	0 (0)	0 (0)	0 (0)	
ACL	0 (0)	0 (0)	6 (54.54)	1 (3.85)	
Anti B2GP	1 (3.70)	0 (0)	8 (72.72)	1 (3.85)	
RF	1 (3.70)	11 (47.83)	0 (0)	3 (11.54)	
C3c mg/dL, mean ± SD	82.7 ± 21.6	101.8 ± 25.3	100.1 ± 21.2	101.3 ± 28.9	
C4 mg/dL, mean ± SD	14.6 ± 7.2	19.1 ± 8.8	16.9 ± 9.1	16.6 ± 6.2	
Abnormal Liver function, *n* (%)	4 (14.81)	1 (4.35)	0 (0)	2 (7.69)	-
GERD, *n* (%)	5 (18.51)	11 (47.83)	1 (9.09)	10 (38.46)	-
Pericarditis, *n* (%)	4 (14.81)	0 (0)	0 (0)	0 (0)	-
Hypertension, *n* (%)	6 (22.22)	2 (8.69)	2 (18.18)	5 (19.23)	-
Valve lesions, *n* (%)	1 (3.70)	1 (4.35)	1 (9.09)	1 (3.85)	-
Dyslipidemia, *n* (%)	5 (18.51)	2 (8.69)	3 (27.27)	3 (11.54)	-
Abnormal creatinine, *n* (%)	7 (25.92)	1 (4.35)	2 (18.18)	3 (11.54)	-
Abnormal urine, *n* (%)	10 (37.03)	1 (4.35)	3 (27.27)	2 (7.69)	-
Proteinuria, *n* (%)	7 (25.92)	1 (4.35)	3 (27.27)	0 (0)	-
Anemia past, *n* (%)	3 (11.1)	1 (4.35)	0 (0)	0 (0)	-
Low platelet count, *n* (%)	8 (29.62)	2 (8.69)	2 (18.18)	1 (3.85)	-
Low WBC, *n* (%)	18 (66.67)	6 (26.09)	2 (18.18)	7 (26.92)	-
Pleuritis, *n* (%)	1 (3.70)	1 (4.35)	0 (0)	0 (0)	-
Arthritis, *n* (%)	17 (62.96)	3 (13.04)	1 (9.09)	6 (23.08)	-
Myopathy, *n* (%)	2 (7.4)	0 (0)	0 (0)	0 (0)	-
CNS involvement, *n* (%)	3 (11.1)	1 (4.35)	4 (36.36)	0 (0)	-
PNS involvement, *n* (%)	0 (0)	0 (0)	2 (18.18)	1 (3.85)	-
Mucositis, *n* (%)	10 (37.03)	0 (0)	2 (18.18)	4 (15.38)	-
Cutaneous active lupus, *n* (%)	19 (70.37)	1 (4.35)	1 (9.09)	2 (7.69)	-
Cutaneous chronic lupus, *n* (%)	5 (18.51)	0 (0)	1 (9.09)	4 (15.38)	-
Photosensitivity, *n* (%)	22 (81.48)	2 (8.69)	1 (9.09)	13 (50)	-
Puffy fingers, *n* (%)	2 (7.4)	0 (0)	0 (0)	0 (0)	-
Sicca, *n* (%)	12 (44.44)	21 (91.30)	1 (9.09)	9 (34.62)	-
Inflammation, *n* (%)	17 (62.96)	10 (43.48)	1 (9.09)	13 (50)	-
PGA, mean (SD)	28.52 (21.61)	30.78 (18.97)	47.27 (29.44)	30.28 (18.2)	-
Fever, *n* (%)	0 (0)	2 (8.69)	1 (9.09)	2 (7.69)	-
Hypergammaglobulinemia, *n* (%)	12 (44.44)	12 (52.17)	0 (0)	9 (34.62)	-
Venous thrombosis, *n* (%)	2 (7.4)	0 (0)	6 (54.54)	1 (3.85)	-
Raynaud’s phenomenon, *n* (%)	8 (29.62)	2 (8.69)	1 (9.09)	11 (42.3)	-
Miscarriage, *n* (%)	2 (7.4)	1 (4.35)	7 (63.63)	0 (0)	-
Statin use, *n* (%)	2 (7.4)	2 (8.69)	1 (9.09)	2 (7.69)	-
Prednisone (>5 mg/day), *n* (%)	7 (25.92)	3 (13.04)	1 (9.09)	9 (34.62)	-
Prednisone dose, mean mg/day	7.55	8.62	7	5.67	-
HCQ use, *n* (%)	19 (70.37)	9 (39.13)	8 (72.72)	13 (50)	-
Immunosuppressant use, *n* (%)	10 (37.04)	2 (8.69)	0 (0)	3 (11.54)	-

Note: SLE, systemic lupus erythematosus; SjS, Sjogren’s syndrome; PAPS, primary antiphospholipid syndrome; UCTD, undifferentiated connective tissue disease; HC, healthy controls; SD, standard deviation. ANA, anti-nuclear antibodies; anti-Ro60/SSA and anti-La/SSB, anti-Sjögren’s-syndrome-related antigen A and B; anti dsDNA, anti-double stranded DNA; anti Sm, anti-Smith antibodies; ACL, anti-cardiolipin antibodies; anti B2GP, anti-beta2glicoprotein-I antibodies; RF, rheumatoid factor; GERD, gastro-esophageal reflux; WBC, white blood cells; CNS, central nervous system; PNS, peripheral nervous system; PGA, physician’s global assessment scale; HCQ, hydroxychloroquine. For clinical data the occurrence of symptom in the medical history (“ever”) is counted as an entry. For therapies, the current use is listed.

## Supplementary Materials

The following are available online at https://www.mdpi.com/2077-0383/8/9/1291/s1, Figure S1: Alpha diversity, Figure S2: Beta diversity, Figure S3: Performance of microbial genera in classification tasks after internal validation and feature selection, Figure S4: Selected genera abundance in systemic autoimmune diseases, Figure S5: Selected genera abundant in healthy controls, Figure S6: Performance of metabolomic peaks in classification tasks after internal validation and feature selection, Figure S7: Genera x metabolites cross-correlation, Table S1: Relative abundance of selected bacterial genera in in healthy controls (HCs) and patients with systemic autoimmune diseases (SADs), Table S2: Selected metabolites in healthy controls (HCs) and patients with systemic autoimmune diseases (SADs).
