# Supplementary material for: Identification of a Shared Microbiomic and Metabolomic Profile in Systemic Autoimmune Diseases
Source: J Clin Med. 2019 Aug 23;8(9):1291. doi: 10.3390/jcm8091291 (PMC6780636; doi:10.3390/jcm8091291)
Supplement: Supplementary file 1 [file jcm-08-01291-s001.zip › jcm-562360-supplemental/Supplemental Material.docx.doc]

**Supplemental Figure 1 – Alpha diversity**

Alpha diversity by Chao and Shannon index showing no differences after Kruskal-Wallis test.

**Supplemental Figure 2 – Beta diversity**

Results from principal coordinate analysis (PCoA) showing a high degree of overlap of the studied groups.

**Supplemental Figure 3 – Performance of microbial genera in classification tasks after internal validation and feature selection.**

Performance of selected *genera* in classifying the different classes (diseases/healthy controls) according to three fitness measures. Values represent the mean of 10 x 5-fold cross-validation runs with 5-fold nested cross-validation to select the *genera* that globally maximize the balanced accuracy (BA) of all the pairwise comparisons. Values close to 1 indicate perfect fit, while values close to 0.5 indicate random choice (inability to discriminate classes).

**Supplemental Figure 4 – Selected genera abundance in systemic autoimmune diseases**

Boxplots/Scatterplots of selected genera whose abundance was statistically different among groups (after Kruskal-Wallis test and false discovery rate correction), and higher in systemic autoimmune diseases than in healthy controls (HC). *Post-hoc* analysis after Dunn’s test is also reported.

The taxon relative abundance is reported after cube-transformation.

**Supplemental Figure 5 – Selected genera abundant in healthy controls**

Boxplots/Scatterplots of selected genera whose abundance was statistically different among groups (after Kruskal-Wallis test and false discovery rate correction), and lower in systemic autoimmune diseases than healthy controls (HC). *Post-hoc* analysis after Dunn’s test is also reported.

The taxon relative abundance is reported after cube-transformation.

**Supplemental Figure 6 – Performance of metabolomic peaks in classification tasks after internal validation and feature selection.**

Performance of selected *metabolites* in classifying the different classes (diseases/healthy controls) according to three fitness measures. Values represents the mean of 10x 5-fold cross-validation runs with 5-fold nested cross-validation to select the *metabolites* that globally maximize the balanced accuracy (BA) of all the pairwise comparisons. Values close to 1 indicate perfect fit, while values close to 0.5 indicate random choice (inability to discriminate classes).

**Supplemental Figure 7 - Genera x metabolites cross-correlation**

Refer to high-resolution image for viewing.

Cross-correlation between metabolites and microbial genera after correction for confounding covariates and statistical correction after permutation testing. Significant correlations at the 0.05 threshold are highlighted. Diamonds, positive correlations, rounds negative correlations.

Metabolites (x-axis) and bacterial general (y-axis) are clustered according to the partial correlation r values.

Red-arrows indicate butyrate-producing bacteria.

**Supplemental Table 1 – Relative abundance of selected bacterial genera in in healthy controls (HCs) and patients with systemic autoimmune diseases (SADs)**

| **Genus** | **HCs** | **SADs** | **PAPs** | **SLE** | **SjS** | **UCTD** |
| --- | --- | --- | --- | --- | --- | --- |
| Bifidobacterium | 0.49 ± 0.95% | 2.67 ± 4.09% | 5.85 ± 5.87% | 2.42 ± 3.86% | 2.68 ± 4.61% | 1.6 ± 2.07% |
| Collinsella | 0.12 ± 0.15% | 0.47 ± 0.81% | 1.26 ± 1.18% | 0.61 ± 0.93% | 0.05 ± 0.05% | 0.36 ± 0.58% |
| Um_Coriobacteriaceae_family | 0.02 ± 0.03% | 0.15 ± 0.25% | 0.34 ± 0.49% | 0.09 ± 0.12% | 0.16 ± 0.25% | 0.12 ± 0.17% |
| Bacteroides | 26.95 ± 17.26% | 21.69 ± 16.06% | 17.09 ± 13.55% | 22.87 ± 16.53% | 19.06 ± 15.9% | 24.67 ± 16.83% |
| Prevotella7 | 1.88 ± 6.37% | 0.01 ± 0.09% | 0 ± 0% | 0 ± 0% | 0.04 ± 0.17% | 0.01 ± 0.02% |
| Um_Prevotellaceae_family | 1.19 ± 4.92% | 0.06 ± 0.17% | 0 ± 0% | 0.01 ± 0.02% | 0.14 ± 0.27% | 0.07 ± 0.16% |
| Alistipes | 4.07 ± 3.84% | 5.44 ± 5.55% | 5.16 ± 6.3% | 5.75 ± 5.95% | 7.08 ± 5.74% | 3.81 ± 4.38% |
| Dorea | 0.16 ± 0.21% | 0.78 ± 1.84% | 2.36 ± 4.59% | 0.68 ± 0.93% | 0.33 ± 0.55% | 0.61 ± 0.97% |
| Eubacterium_eligens_group | 1.75 ± 1.83% | 0.75 ± 1.56% | 0.17 ± 0.17% | 0.46 ± 0.57% | 0.45 ± 0.64% | 1.54 ± 2.58% |
| Lachnoclostridium | 0.92 ± 1.81% | 0.07 ± 0.09% | 0.03 ± 0.03% | 0.06 ± 0.08% | 0.07 ± 0.1% | 0.09 ± 0.11% |
| Lachnospiraceae_ND3007_group | 0.14 ± 0.23% | 0.03 ± 0.04% | 0.02 ± 0.02% | 0.02 ± 0.03% | 0.04 ± 0.05% | 0.04 ± 0.05% |
| Lachnospiraceae_NK4A136_group | 0.47 ± 0.51% | 0.62 ± 1.07% | 0.41 ± 0.51% | 0.94 ± 1.68% | 0.57 ± 0.49% | 0.41 ± 0.7% |
| Lachnospiraceae_NK4B4_group | 0.03 ± 0.06% | 0.01 ± 0.01% | 0 ± 0.01% | 0.01 ± 0.02% | 0.01 ± 0.01% | 0.01 ± 0.02% |
| Lachnospira | 1.99 ± 2.07% | 0.18 ± 0.44% | 0.04 ± 0.04% | 0.14 ± 0.25% | 0.34 ± 0.76% | 0.14 ± 0.27% |
| Marvinbryantia | 0 ± 0% | 0.05 ± 0.2% | 0 ± 0% | 0 ± 0.02% | 0.13 ± 0.25% | 0.05 ± 0.27% |
| Um_Ruminococcaceae_family | 0.73 ± 1.08% | 0.74 ± 0.68% | 0.66 ± 0.41% | 0.66 ± 0.66% | 0.94 ± 0.98% | 0.7 ± 0.44% |
| Faecalibacterium | 10.4 ± 10.58% | 6.61 ± 6.85% | 5.52 ± 4.48% | 7.15 ± 8.38% | 7.38 ± 7.89% | 5.83 ± 4.88% |
| Oscillospira | 0.46 ± 0.53% | 0.54 ± 0.73% | 0.63 ± 0.93% | 0.33 ± 0.46% | 0.47 ± 0.48% | 0.79 ± 0.97% |
| Ruminiclostridium5 | 0.09 ± 0.1% | 0.37 ± 0.56% | 0.2 ± 0.14% | 0.54 ± 0.92% | 0.29 ± 0.3% | 0.34 ± 0.25% |
| Ruminococcaceae_UCG_005 | 0.74 ± 0.68% | 0.58 ± 0.79% | 0.68 ± 0.92% | 0.48 ± 0.7% | 0.78 ± 0.77% | 0.48 ± 0.85% |
| Ruminococcaceae_UCG_013 | 0.12 ± 0.13% | 0.44 ± 0.63% | 0.53 ± 0.8% | 0.26 ± 0.32% | 0.6 ± 0.59% | 0.47 ± 0.79% |
| Turicibacter | 0.04 ± 0.07% | 0.2 ± 1.15% | 0.06 ± 0.09% | 0.51 ± 2.03% | 0.01 ± 0.02% | 0.09 ± 0.22% |
| Streptococcus | 0.13 ± 0.21% | 1.18 ± 2.71% | 0.35 ± 0.35% | 2.22 ± 4.25% | 0.91 ± 1.87% | 0.66 ± 1.24% |
| Dialister | 0.99 ± 1.75% | 3.89 ± 8.11% | 1.41 ± 2.03% | 2.5 ± 4.78% | 5.98 ± 9.54% | 4.54 ± 10.59% |
| Veillonella | 0.02 ± 0.03% | 0.15 ± 0.32% | 0.02 ± 0.02% | 0.22 ± 0.44% | 0.11 ± 0.2% | 0.17 ± 0.3% |
| Victivallis | 0.08 ± 0.24% | 0 ± 0% | 0 ± 0% | 0 ± 0% | 0 ± 0% | 0 ± 0.01% |
| Sutterella | 1.1 ± 1.1% | 0.2 ± 0.47% | 0.07 ± 0.12% | 0.2 ± 0.46% | 0.12 ± 0.29% | 0.32 ± 0.65% |
| Um_Enterobacteriaceae_family | 0.21 ± 0.92% | 0.06 ± 0.1% | 0.06 ± 0.06% | 0.07 ± 0.11% | 0.07 ± 0.13% | 0.03 ± 0.04% |
| Taxa_below1p | 1.04 ± 0.58% | 1.62 ± 0.81% | 1.26 ± 0.48% | 1.53 ± 0.83% | 1.93 ± 0.91% | 1.58 ± 0.75% |

Relative abundance of *genera* that in data mining models allow the best discrimination between HCs and SADs, including primary antyphospholipid syndrome (PAPs), systemic lupus erythematosus (SLE), primary Sjogren’s syndrome (SjS) and undifferentiated connective tissue disease (UCTD). Values expressed as mean ± standard deviation excluding 5% of outliers. Genera whose abundance is statistically different among groups are represented in Supplemental figures 4 and 5.

**Supplemental Table 2 – Selected metabolites in healthy controls (HCs) and patients with systemic autoimmune diseases (SADs)**

| **Peak** | **Formula** | **Compound** | **HCs** | **SADs** | **PAPs** | **SLE** | **SjS** | **UCTD** |
| --- | --- | --- | --- | --- | --- | --- | --- | --- |
| 79 | C10H20O7P2 | Geranyl diphosphate | 9040753.4 ± 682151.8 | 8411418.3 ± 868036.3 | 8184454.8 ± 865208.3 | 8328571.3 ± 837503.6 | 8534270.6 ± 980149.9 | 8506269.3 ± 828486.2 |
| 194 | C44H80NO8P | PC(36:4) | 7470755.7 ± 4409552.3**^b^** | 3735103.9 ± 3324809.9 | 2337792.3 ± 456560.3 | 2815871.3 ± 2614945.5 | 4700893.5 ± 3525093 | 4604850.4 ± 4161578.9 |
| 135 | C26H50NO7P | LysoPC(18:2) | 2135437.7 ± 833017.9**^b,d^** | 1489609.8 ± 530189.3 | 1586749.2 ± 306092.2 | 1506966.4 ± 654761.1 | 1261367.6 ± 504115.9 | 1615763.1 ± 433460.4 |
| 173 | C39H77N2O6P | SM(d18:1/16:1) | 2039121.1 ± 346731.5 | 2088453 ± 465706.5 | 2028568.5 ± 341487.8 | 1928386.1 ± 333047.2 | 2419564.5 ± 498016.3 | 2020049.2 ± 500817.2 |
| 60 | C8H14N2O6 | L-beta-aspartyl-L-threonine | 1374947.9 ± 95519.3**^d^** | 1269851.3 ± 155489.6 | 1292013.7 ± 96531.7 | 1292316 ± 139591.4 | 1187759.9 ± 205163.1 | 1302830.4 ± 128345 |
| 168 | C37H75N2O6P | SM(32.1) | 1318674.6 ± 229323.6 | 1295243.1 ± 367665.6 | 1280224.5 ± 303815.2 | 1174147.7 ± 290508.2 | 1522351.9 ± 418529.4 | 1249101.5 ± 362531.1 |
| 38 | C9H11NO3 | L-Tyrosine | 1231718.8 ± 357192.9 | 1181304.3 ± 282546.4 | 956994.5 ± 115665.3 | 1173854 ± 286611.4 | 1218014.8 ± 246986 | 1261902.6 ± 316226.6 |
| 3 | C26H54NO7P | LysoPC(18:0) | 896064.9 ± 148810.6 | 800652.3 ± 233858.9 | 887655 ± 188211.7 | 796368 ± 272015.3 | 750437.1 ± 202746.5 | 807441.8 ± 231773.7 |
| 225 | C48H74NO8P | PE-NMe(42:11) | 771707.9 ± 158427.9 | 808434.5 ± 217921.8 | 727021 ± 184973 | 750871.4 ± 158184 | 959614.2 ± 249785.3 | 784524.4 ± 212162 |
| 192 | C47H38NO8P | PC(34:3) | 724292.3 ± 212919.8**^a,b,c^** | 1218494.9 ± 535495.9 | 1244577.2 ± 323166.1 | 1078529.9 ± 410703.6 | 1637073.3 ± 636516.6**^b,d^** | 1015185.7 ± 469353.3 |
| 81 | C20H30O2 | Eicosapentaenoic acid | 638128.6 ± 455321.7**^a^** | 914968.9 ± 583356.5 | 1398512.8 ± 714190.4 | 761761.3 ± 468912 | 1057763.6 ± 598014.5 | 746707.6 ± 496043 |
| 29 | C7H15NO3 | L-Carnitine | 506683.7 ± 149241.7 | 492874.9 ± 206429.7 | 525550.4 ± 171307.5 | 506617.2 ± 196596.2 | 522752.2 ± 202238 | 437541 ± 234800.7 |
| 220 | C47H91N2O6P | SM(d18:2/24:1) | 414710.2 ± 88170.4 | 358066 ± 169283.2 | 253651.2 ± 194630.6 | 321274.7 ± 141025.3 | 412977 ± 206327 | 401554 ± 124111.5 |
| 193 | C42H80NO8P | PC (34:2) | 406990 ± 29941.7 | 412862.4 ± 43755.9 | 416937.6 ± 32289.6 | 405714.7 ± 41537 | 426647.6 ± 64183.7 | 407548.3 ± 25947 |
| 7 |  | Unknown | 349812.2 ± 37940.4 | 358033.9 ± 70200.3 | 389851.3 ± 50057.3 | 353551.9 ± 61058.7 | 368577.9 ± 73646.4 | 339706.6 ± 81339.3 |
| 178 | C42H80NO7P | PC(18:2) | 312236.3 ± 102083.2 | 348072.9 ± 119215.7 | 376769.6 ± 82906.1 | 312881.6 ± 108535 | 381869.4 ± 113283.6 | 346346.7 ± 142485.8 |
| 133 | C25H52NO7P | LysoPC(17:0) | 302792.4 ± 140888.8 | 263931.3 ± 89996.3 | 281662.4 ± 86194.6 | 269839.4 ± 115863.9 | 261379.8 ± 70421.2 | 251284.3 ± 75216.3 |
| 121 | C24H50NO6P | LysoPC(P-16:0) | 279111 ± 102426.2 | 268175.9 ± 95683.9 | 340026.3 ± 65428.8 | 264575.7 ± 105454.1 | 253410.1 ± 79190.6 | 251599.4 ± 98333.9 |
| 228 |  | Unknown | 248885.4 ± 51517 | 267245.1 ± 55194 | 298564.9 ± 37169.2 | 258906.6 ± 61097.8 | 270462.8 ± 51644.4 | 259589.7 ± 55532.1 |
| 171 | C37H73N2O6P | SM(d18:2/14:0) | 247997.3 ± 65215.5 | 231913.4 ± 103989 | 211531.4 ± 69077.9 | 196191.3 ± 63002.2 | 307758.5 ± 137198.3**^b,d^** | 218238.3 ± 95276.8 |
| 117 | C29H45NO4 | CervonylCarnitine**^a^** | 245678.2 ± 177720.6 | 178147.4 ± 310192.5 | 64490.5 ± 39210.6 | 153147.7 ± 98801.8 | 275360.2 ± 597443.5 | 177354.2 ± 131009.7 |
| 123 | C24H52NO6P | PC(O-16:0/0:0) | 241258.5 ± 111188.9 | 235361 ± 81517.6 | 303834.6 ± 91508.2 | 246713 ± 81860.3 | 190491.7 ± 57463.5 | 228597.5 ± 72571.6 |
| 164 |  | Unknown | 224805.4 ± 29875.9**^a,b,c,d^** | 160322.4 ± 47693.2 | 133529.5 ± 32345.4 | 161953.2 ± 45994.6 | 165930.9 ± 43376.7 | 166094 ± 56581.9 |
| 269 |  | Unknown | 214149 ± 45583.7**^a b,d^** | 163149.9 ± 50699.8 | 139575.5 ± 26936 | 148070.4 ± 43551.1 | 185998.7 ± 51572.1 | 171878.5 ± 57703.1 |
| 196 | C42H82NO8P | PC(34:1) | 202436.1 ± 74509 | 213627.6 ± 92933.4 | 188638.2 ± 92517.7 | 202910.4 ± 80188.8 | 268709.7 ± 95497.7 | 191236.2 ± 91086.3 |
| 204 | C44H80NO8P | PC(36:4) | 201407.6 ± 45188.7 | 187728.9 ± 50104.3 | 182064.3 ± 38307.5 | 176267 ± 51171.2 | 207898.5 ± 52958.5 | 186411.9 ± 49000.9 |
| 108 | C26H36O4 | Boviquinone 4 | 174730.8 ± 59310.2 | 196422.1 ± 102298.6 | 203021.2 ± 83378.3 | 199767 ± 130181.5 | 208259 ± 101829.5 | 179770.5 ± 75257.5 |
| 110 | C25H47NO4 | Octadecenoylcarnitine | 157547.9 ± 106381.5**^a,b^** | 216974.3 ± 100948.4 | 257640.7 ± 105009.1 | 230903.5 ± 107936.3 | 193926.4 ± 96154.1 | 201871.9 ± 92564.2 |
| 157 | C35H43NO8 | Taxine B | 139779.2 ± 25410.3 | 140848.3 ± 23520.1 | 144373.9 ± 12315.1 | 143666.6 ± 29893 | 134293.4 ± 20903.9 | 141524.4 ± 21401.8 |
| 129 | C20H50NO3 | Unknown | 111418.6 ± 15589.9**^a,b,c,d^** | 94182.3 ± 23900.7 | 90643.5 ± 14298.9 | 101371 ± 19669.7 | 78598.6 ± 23146.1 | 100703.5 ± 26846.4 |
| 215 |  | Unknown | 103929.7 ± 25981.3 | 116557.3 ± 27680.1 | 109656.1 ± 19886.6 | 108474.6 ± 21661.4 | 135825.4 ± 34424.5 | 112756.7 ± 24075.4 |
| 156 |  | Unknown | 103157.2 ± 117517.9**^a^** | 180962.3 ± 167952.8 | 326863 ± 232639.7 | 135744.4 ± 117657.8 | 220493.4 ± 174217.1 | 132018.9 ± 136562.2 |
| 36 | C7H8N4O2 | Theobromine/Paraxanthine/Theophylline | 89057.3 ± 55143.8 | 83883.5 ± 65367.8 | 79130 ± 47908.9 | 78140.2 ± 64887.2 | 79693.4 ± 38150.4 | 96015.1 ± 88713.2 |
| 238 |  | Unknown | 87189.9 ± 46948.6**^a,c^** | 115863.5 ± 34047.4 | 132037.4 ± 23791.6 | 108968.3 ± 28615.3 | 134512.2 ± 25551.4 | 100667.1 ± 40584.8 |
| 92 |  | Unknown | 74626.1 ± 15622.8 | 78963.3 ± 30535.5 | 62557.7 ± 7472.3 | 81429.9 ± 39798.7 | 87907.6 ± 30794 | 76254.2 ± 21736.6 |
| 93 | C19H37NO4 | Dodecanoylcarnitine | 69372.4 ± 50378.3 | 74989.8 ± 52946.6 | 74161.4 ± 45266.8 | 61982.4 ± 40937.9 | 76106.7 ± 44957.5 | 89072 ± 70785.5 |
| 28 | C6H9NO4 | N-methyl-2-oxoglutaramate | 68370.6 ± 25372.2 | 66938.6 ± 31643.7 | 72979.1 ± 24140.9 | 70164.4 ± 37925.6 | 70960.5 ± 29268.6 | 57189.5 ± 28221.8 |
| 118 |  | Unknown | 60471.9 ± 5326.2 | 57620.8 ± 6364 | 56812.9 ± 4145.6 | 58334.5 ± 7304.1 | 58010.8 ± 6316.4 | 56863.1 ± 6344.5 |
| 52 | C11H6O5 | 4.8-dihydroxy-2H-furo[2.3-h]chromen-2-one | 59879.9 ± 10222.1 | 52155.5 ± 9621.2 | 49453.9 ± 6774.2 | 52043.7 ± 8873.2 | 52556.1 ± 13579.7 | 53185.5 ± 7742.9 |
| 63 | C12H23NO4 | IsovalerylCarnitine | 59288.6 ± 28681.3 | 49565.3 ± 23235.3 | 44606.5 ± 14646.5 | 47103.9 ± 25930.2 | 47066.2 ± 20009.2 | 56689.8 ± 25297.4 |
| 65 |  | Unknown | 27785.3 ± 2667.8 | 27521.2 ± 4042.6 | 26620.7 ± 1645.5 | 27441.4 ± 3590.5 | 28984.9 ± 5135.6 | 26803.9 ± 4149.9 |

Peak areas of metabolites that in data mining models allow the best discrimination between HCs and SADs, including primary antyphospholipid syndrome (PAPs), systemic lupus erythematosus (SLE), primary Sjogren’s syndrome (SjS) and undifferentiated connective tissue disease (UCTD). Values expressed as mean ± standard deviation excluding 5% of outliers. a, p<0.05 vs PAPs; b, p<0.05 vs SLE; c, p<0.05 vs SjS; d, p<0.05 vs UCTD; pairwise comparisons conducted with Dunn’s test when Kruskal-Wallis test was significant at the 0.05 false discovery rate threshold.
